# The Importance of Religion and Spirituality in Cultural Psychiatry: Reply to Drs Persad and Oyewumi

**DOI:** 10.1177/07067437221087939

**Published:** 2022-03-31

**Authors:** Laurence J. Kirmayer, Lisa Andermann, Kenneth Fung, Jaswant Guzder, G. Eric Jarvis

**Affiliations:** 1 Division of Social & Transcultural Psychiatry, 12367 McGill University, Montreal, Quebec, Canada,; 2 Department of Psychiatry, 12366 University of Toronto, Toronto, Ontario, Canada

We appreciate the response to the CPA Position Paper on Training in Cultural Psychiatry from Drs. Persad and Oyewumi.^1^ We completely concur with their comments underscoring the importance of religion and spirituality in cultural psychiatry. As they note, although the guidelines identify religion and spirituality as important aspects of cultural identity and key topics in the training curriculum, we did not give this a central place in our discussion of the many dimensions of culture. This mainly reflects space constraints given the broad range of issues that fall under the rubric of culture that we sought to include.

Religion and spirituality can extend integrative approaches like the biopsychosocial model with another level of analysis relevant to understanding etiology, treatment, and prevention. Religion is central to identity for many people and a source of meaning, values, purpose, community, collective identity, and coping resources in times of illness and adversity.^2^ Religious systems provide frameworks through which mental health symptoms and problems are interpreted in ways that guide individual and family coping as well as the larger social responses of social support and integration or stigmatization and marginalization. While religious participation generally is positively correlated with mental health and well-being,^3^ it can also be a source of conflict in some instances.^4^ Religious groups also continue to be targets of prejudice and discrimination. Attention to religion is particularly important in global mental health because, in many countries, religious institutions and practitioners may provide an important path of help-seeking for people with mental health problems. Collaboration with religious and spiritual leaders, teachers, and helpers can provide a valuable resource in mental health care. For all these reasons, it is important to recognize individuals’ relationships to systems of religion and spirituality in assessment and, when appropriate for the individual, mobilize these connections in providing care.

Although the DSM-5 Cultural Formulation Interview includes a supplementary module on religion and spirituality, religion has been a relatively neglected topic in psychiatric research and training. In part, this may be a legacy of psychoanalytic views and a reflection of the secularization of some societies. More recently, the politicization of religious identity and the assertion of restrictive forms of secularism in part of Canada may be contributing to increases in discrimination against some groups^5^ and pose challenges to multiculturalism and human rights.^6^

The Section on Transcultural Psychiatry has recently completed a position paper on *Taking Action on Racism and Social Justice in Psychiatry*. In future work, the Section plans to prepare another paper on the importance of religion in psychiatry. This will discuss the ways in which attention to spirituality and religion can be integrated as a key element in person-centered psychiatry.
